# Physiological Sensors Based Emotion Recognition While Experiencing Tactile Enhanced Multimedia

**DOI:** 10.3390/s20144037

**Published:** 2020-07-21

**Authors:** Aasim Raheel, Muhammad Majid, Majdi Alnowami, Syed Muhammad Anwar

**Affiliations:** 1 Department of Computer Engineering, University of Engineering and Technology, Taxila 47050, Pakistan; asim.raheel@uettaxila.edu.pk; 2 Department of Nuclear Engineering, King Abdulaziz University, Jeddah 21589, Saudi Arabia; malnowaimi@kau.edu.sa; 3 Department of Software Engineering, University of Engineering and Technology, Taxila 47050, Pakistan; s.anwar@uettaxila.edu.pk

**Keywords:** emotion recognition, wearable sensors, tactile enhanced multimedia, physiological signal processing, classification

## Abstract

Emotion recognition has increased the potential of affective computing by getting an instant feedback from users and thereby, have a better understanding of their behavior. Physiological sensors have been used to recognize human emotions in response to audio and video content that engages single (auditory) and multiple (two: auditory and vision) human senses, respectively. In this study, human emotions were recognized using physiological signals observed in response to tactile enhanced multimedia content that engages three (tactile, vision, and auditory) human senses. The aim was to give users an enhanced real-world sensation while engaging with multimedia content. To this end, four videos were selected and synchronized with an electric fan and a heater, based on timestamps within the scenes, to generate tactile enhanced content with cold and hot air effect respectively. Physiological signals, i.e., electroencephalography (EEG), photoplethysmography (PPG), and galvanic skin response (GSR) were recorded using commercially available sensors, while experiencing these tactile enhanced videos. The precision of the acquired physiological signals (including EEG, PPG, and GSR) is enhanced using pre-processing with a Savitzky-Golay smoothing filter. Frequency domain features (rational asymmetry, differential asymmetry, and correlation) from EEG, time domain features (variance, entropy, kurtosis, and skewness) from GSR, heart rate and heart rate variability from PPG data are extracted. The *K* nearest neighbor classifier is applied to the extracted features to classify four (happy, relaxed, angry, and sad) emotions. Our experimental results show that among individual modalities, PPG-based features gives the highest accuracy of 78.57% as compared to EEG- and GSR-based features. The fusion of EEG, GSR, and PPG features further improved the classification accuracy to 79.76% (for four emotions) when interacting with tactile enhanced multimedia.

## 1. Introduction

Human senses are physiological responses and play a vital role in perception. Humans perceive their surrounding environment using multiple senses, i.e., vision, auditory, gustatory, olfactory, and tactile (touch). The sensing organs transmit information to the human brain, which helps in perceiving the surrounding environment. Traditional multimedia engages only two human senses, i.e., auditory and vision. Whereas the human experience of viewing multimedia content can be enhanced by engaging more than two human senses simultaneously. The multimedia content that can engage more than two human senses simultaneously is termed as multiple sensorial media (mulsemedia) [1,2]. There is a recent focus on mulsemedia with the aim towards providing an immersive real-world environment during multimedia interactions. Mulsemedia could provide a new dimension towards developing immersive systems in diverse fields such as education, medical, advertisement, and home entertainment. Furthermore, recent advancements in wearable sensing technologies have provided a broad spectrum to researchers for analyzing mulsemedia and its impact on human emotions and behavior. A detailed survey of the devices that engage haptic, olfactory, and gustatory senses in addition to vision and hearing for building a mulsemedia environment was presented in [3]. Similarly, a framework was proposed for the delivery of multi-sensory effects to a heterogeneous system [4].

The recognition and adaptation to the affective state of a user have increased the potential of affective computing. Affective state of an individual conveys the emotional intent and is considered as a primary mean of communication. In everyday life, emotions play an essential role in understanding human behavior and non-verbal communication. Emotions are physiological responses evoked in reaction to external stimuli and could be used for evaluating the type of stimulus. Affective computing has augmented the development of models and systems that can process human activity, and in turn simulate it with smart recognition and interpretation [5]. Emotions have been characterized into six basic types including anger, surprise, fear, happiness, and sadness [6] whereas Russell’s Circumplex model categorizes emotions in a two-dimensional space based on the valence and arousal scores [7]. A person’s emotional state may change depending on their subjective experience [8]. An emotional state can be evaluated by varying environmental conditions and this evaluation can benefit from self reports as well as the data collected by various sensing devices [9,10]. Integrating these (sources of information) can help us in better understanding an individual’s behavior or emotional state.

Whenever a person engages with certain emotional stimuli, their feelings are communicated through physiological cues like brain activity, heart rate, facial expressions, body gestures, or change in vocals. These cues are used in associating the emotional state of an individual with an external stimulus. Emotion recognition using speech [11,12,13], facial expressions [14,15,16] and their fusion [17,18] has been explored. These conventional methods for emotion recognition have limitations such as privacy and camera positioning [19]. Emotion recognition from physiological cues like brain activity, skin conductance, and heart rate has shown promising results and is relatively new in this line of research. Human emotions are generated from the limbic system, which directs our attention and effects brain patterns [20,21]. Recently, the interest in brain activity evaluation using electroencephalography (EEG) has increased due to the availability of low-cost wearable headsets and their easy usage. Emotional markers are present in EEG signals, which cannot be easily deceived by a user’s voluntary actions [22,23]. Emotion recognition using EEG focuses on identifying the emotional state of the mind. The changes in skin conductance are also observed during differential emotional states [24,25]. A variation in heart rate has been reported as a discriminating cue for human emotion recognition [26,27].

The quality of experience (QoE) of mulsemedia content has been subjectively analyzed where different genders and age groups have shown a varying level of perception [28]. Similarly, synchronization errors in audio-visual content and external devices have been analyzed and discussed to enhance the experience level of viewers [29]. Mulsemedia has been explored in a 360-degree video environment, where a higher quality of perception and enjoyment was achieved [30]. The QoE of mulsemedia has been objectively analyzed using heart rate and electrodermal activity (EDA) [31]. A correlation was found between these objective metrics with the arousal and subjective ratings of QoE [32]. Eye gaze data and heart rate have been analyzed for evaluating the enjoyment and perception of viewers while experiencing mulsemedia content [33,34]. Cross-modal correspondences were also identified when mapped with multi-sensory effects. Eye gaze and heart rate have a significant influence on QoE of viewers while experiencing cross-modal sensory effects. Human emotions were recognized in response to tactile enhanced multimedia (TEM) using brain signals [35]. EEG data were acquired and four emotions (i.e., sad, relaxed, angry, and happy) were classified using time domain features. A significant change in human emotions was observed by engaging an additional tactile sense. An increase in emotion recognition accuracy was achieved by extracting frequency domain features [36].

Hence, the human response to mulsemedia content can be evaluated using various physiological signals. While TEM clips (for stimuli) and EEG data were used for recognizing emotions [35,36], there is no multimodal physiological signals based emotion recognition framework that has used TEM as stimulus. Towards this, we generate four TEM clips and curate a multimodal dataset based on EEG, galvanic skin response (GSR), and photoplethysmography (PPG) signals in response to TEM clips. Emotion annotation is achieved using self-assessment manikin (SAM) questionnaire. Four human emotions (sad, relaxed, happy, and angry) are recognized using each modality (individually) and fusion of these modalities. Our results show that the fusion strategy achieves better performance for emotion recognition. Our major contributions in this work are two-fold i.e.,

1. We present a method, utilizing multi-modal physiological signals including EEG, GSR, and PPG (acquired using wearable sensors), for emotion recognition in response to TEM.
2. Our results show that utilizing a multimodal fusion strategy for emotion recognition in response to TEM outperforms using data individually from EEG, GSR, and PPG.

The rest of the paper is structured as follows. Section 2 presents the review of latest emotion recognition methods using physiological signals. Section 3 deals with the proposed methodology used for emotion recognition using physiological signals. Emotion recognition results using multiple modalities are presented in Section 4, which is followed by conclusions in Section 5.

## 2. Related Work

In literature, various stimuli have been used to evoke human emotions that engage either a single human sense [37,38,39,40,41] or two human senses [42,43,44,45,46,47,48,49,50]. These evoked emotions are then recognized using features extracted from data acquired using different physiological sensors. Audio music was used as stimuli that engaged a single (i.e., auditory) human sense [37]. EEG based features were extracted to classify human emotions in response to music stimuli and the impact of different genres on different age groups was analyzed. Different nightscape images were used as stimuli, engaging the sense of vision [38]. EEG signals were recorded to analyze brain patterns for evaluating the images in terms of fear. An asymmetry index method was introduced for EEG based emotion recognition in response to images [51]. Different odors were used to recognize emotions using content that engaged the sense of olfaction [39]. EEG signals were used to analyze different brain regions to discriminate pleasant and unpleasant odors. Brain signals were recorded, while engaging the sense of tactile by caressing of textile fabric on the forearm [40]. EEG signals were then used to classify a pleasant and unpleasant state. A practical GSR and PPG based emotion recognition framework was proposed where Geneva affective picture database (GAPED) was used as stimulus [41].

The use of physiological signals is found to be more effective for emotion recognition when compared with speech and gestures [52]. Moreover, multimodal data analysis has a significant impact on emotion detection performance [53,54,55,56,57]. Emotions were recognized by using music as a stimulus [58]. Different physiological signals i.e., EMG, electrocardiogram (ECG), GSR, and respiration changes were acquired to classify different emotional states in the valence-arousal plane. Different time and frequency domain features were extracted and the effectiveness of features was proven by classification accuracies. A music recommendation system was designed by analyzing physiological signals i.e., GSR and PPG [59]. Emotions were linked with the physiological responses in real-time to feed into the recommendation engine. Images were presented, engaging only one human sense, to evoke emotions [60]. Facial expressions and different physiological signals such as GSR, ECG, and temperature data were acquired while presenting the stimulus. A fusion strategy was employed to improve the emotion recognition performance. Different images were presented to detect emotions using EEG and peripheral signals [61]. Emotion detection performance was analyzed by using EEG and peripheral signals individually as well as together.

Different datasets are created for emotion detection using physiological signals in response to various types of stimuli. For instance, dataset for emotion analysis using physiological signals (DEAP) was created to recognize human emotions [42]. Different video clips were displayed to subjects and EEG, GSR, electromyogram (EMG), electrooculogram (EoG), and blood volume pressure (BVP) data were recorded. It was shown that fusing multiple modalities significantly improves emotion recognition performance. Similarly, EEG, EMG, GSR, and temperature data were acquired by presenting video clips as a stimulus [62]. Significant improvement in emotion recognition performance was reported by applying modality fusion strategies. A dataset comprising of EEG, ECG, EoG, and magnetoencephalogram (MEG) signals was created for emotion recognition [63]. Emotions were elicited while presenting musical videos and brain signals were also acquired using MEG sensors and compared with EEG sensors. Another physiological dataset comprising of EEG, ECG, and GSR signals was created to study the effect of personality and emotions by presenting video clips as a stimulus [64]. The relationship between emotions and personality was analyzed using the physiological cues. A physiological dataset was created to study the effect of mood and personality by presenting emotional videos [65]. EEG, GSR, and ECG data were acquired to investigate affective levels using valence and arousal scores. A new multimodal physiological emotion database (MPED) was made public to recognize human emotions using physiological signals including EEG, GSR, respiration, and ECG [66]. The emotions in MPED were categorized based on discrete emotion model. Emotions were recognized by extracting features from ECG and GSR signals [67]. The dataset was acquired by exposing individuals to emotional videos. A pre-processing and feature extraction mechanism was proposed to improve emotion detection accuracy. Emotion detection was performed for ageing people by analyzing ECG, EMG, EDA, and skin temperature data [68]. These physiological responses were analyzed to monitor and detect emotional states in elderly people. These datasets have been created to recognize emotions by analyzing the classifier performance using individual modality or fusion of multiple modalities. Moreover, these studies have presented a stimulus that engages either one human sense (audio music) or two human senses (videos).

The impact of different modalities, i.e., EEG, eye blink, and their fusion on emotion recognition was also investigated [43]. Self-induced emotion patterns were investigated using EEG in response to video clips presented as stimulus [69]. An ensemble classification approach was used to classify emotional states using ECG signals [70]. Emotion monitoring was proposed for healthcare using a low cost wearable EEG headset [71]. Moreover, effect of culture on emotion recognition was investigated using EEG signals by presenting video clips in two different languages [72]. A feature extraction method was proposed to improve emotion recognition accuracy using EEG signals [73]. A quadratic time-frequency feature extraction scheme was proposed to recognize emotions using EEG signals [74]. Physiological signals (EEG and ECG) were used to investigate driver’s emotional states [75]. Emotion recognition was analyzed in response to different movie clips using blood oxygen saturation, GSR, and heart rate variability to evaluate these clips in terms of prompted emotions [44]. The EEG data from DEAP dataset were used and wavelet-based features were extracted from selected channels to recognize emotions [45]. Different frequency bands of brain signals were analyzed to identify more sensitive brain lobes for the emotion recognition task [46]. Physiological and inertial sensors were also used to recognize emotions in response to video clips [47]. EDA, PPG, GSR, accelerometer, skin temperature, blood volume pulse, and heart rate data were collected to recognize different emotional states of an individual. Feature- and decision-level fusion was applied to facial and EEG-based features for multimodal video induced emotion recognition framework [48].

The efficiency of GSR and PPG data from DEAP dataset was examined for emotion categorization. The fusion of GSR and PPG features was also studied to recognize emotions [76]. A machine-learning framework for boredom classification using fusion of EEG and GSR data was proposed in response to videos [77]. A correlation between EEG and GSR data and boredom state was also revealed. Negative emotions were classified using multimodal physiological signals (including ECG, skin temperature, and EDA) in response to videos [78]. ReliefF-based channel selection method was applied to EEG data from DEAP dataset to classify four human emotions [79]. The channel reduction technique was validated by comparing the accuracy and F-score of the system using support vector machine classifier. A commercially available wearable smart bracelet was used to acquire heart rate data while watching traditional video clips to recognize three emotions (neutral, happy, and sad) [80]. Four human emotions, i.e., anger, sadness, joy, and pleasure in response to videos were recognized by extracting four types of features from ECG signals [70]. Ensemble learning methods were employed to improve the classification accuracy of the system for real-world machine learning problems. EEG and GSR signals were also used to classify boredom states in response to video clips [77]. A gradient boosting decision tree (GBDT) based classification scheme was proposed to improve emotion recognition accuracy using physiological signals (ECG, EMG, GSR, and PPG) in response to videos [53]. Fusion of features from EEG and GSR data was used to improve emotion recognition accuracy in response to video clips [81].

Most of the abovementioned emotion recognition methods extract time-, frequency-, and wavelet-domain features from physiological signals. There are some recent studies that have used deep learning techniques for emotion recognition [81,82,83,84,85]. A convolutional neural network (CNN) model was employed to improve emotion recognition performance using physiological signals (including EDA, ECG, and skin temperature) while engaging individuals with video stimulus [86]. A CNN-based model was proposed using DEAP dataset for detecting emotions in response to videos [87]. A capsule network model was proposed using EEG data for emotion recognition [88]. A CNN based model was also proposed to improve accuracy by recognizing emotions using heart rate variability and respiration changes [85]. A deep belief network was proposed for EEG-based emotion recognition, which selected critical frequency bands and channels [82]. Spatial temporal recurrent neural network was proposed for emotion recognition task and showed promising results on EEG and facial expression dataset [83]. EEG and GSR data from DEAP were used to improve the emotion classification accuracy [81]. Spectrogram calculated from EEG signals was given as input to the CNN to extract EEG features, which was then fused with GSR based features. Another CNN-based approach was proposed to recognize emotions in response to videos and results were tested in a subject-dependent and subject-independent manner [84]. Six basic emotions were classified using various CNN models in response to videos as stimuli [85]. Although, high classification accuracy was achieved for selective CNN models, training these deep CNN models remains a challenge. A comprehensive review of emotion recognition and sentiment analysis using multimodal data was presented in some recent works [10,22,89].

Recently, emotion recognition techniques have been explored in response to content engaging three human senses (mulsemedia) [35,36,90,91]. Olfaction enhanced multimedia engaging sense of vision, olfaction, and auditory was generated [90]. Brain activity was statistically analyzed and it was reported that by engaging olfactory sense with traditional multimedia significantly activates different brain regions. Features from these brain regions were utilized to recognize pleasantness states and it was identified that the olfaction enhanced content recognizes human emotions more accurately as compared to traditional multimedia. A vibro-tactile enhanced multimedia was used as stimulus that engaged the sense of vision, auditory, and tactile [91]. Heart rate and eye-tracking data were used to analyze the effect of vibro-tactile enhanced multimedia on user’s perception. Two TEM clips were used as stimuli and EEG signals were used to recognize four human emotions [35,36]. A summary of recent works on emotion recognition using physiological signals is presented in Table 1. It should be noted that these methods are delineated based on stimuli (including images and videos). While for videos, a significant emotion classification accuracy (>90%) has been reported in multiple instances, but for TEM the performance has been significantly lower. In this work, not only the number of TEM clips is increased but also multimodal strategy for emotion recognition in response to TEM is proposed.

## 3. Proposed Methodology

**Figure 1:**
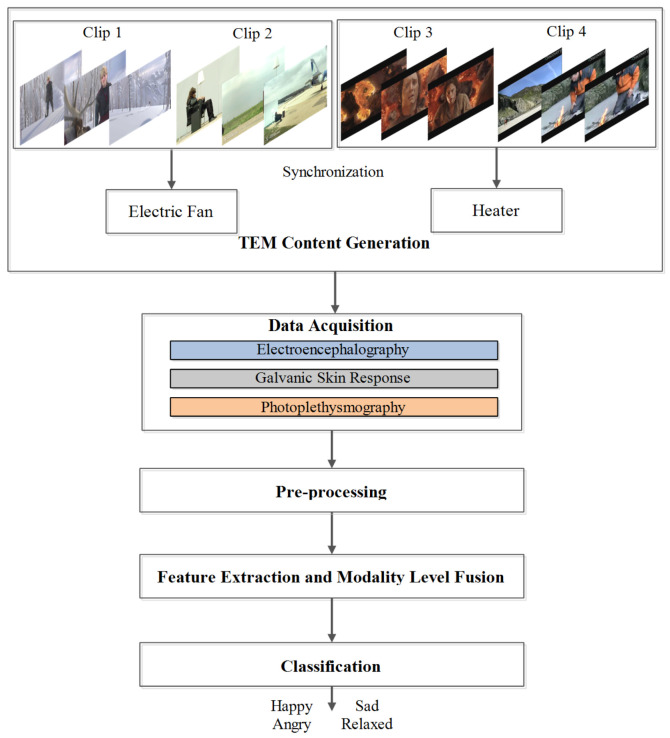
Our proposed methodology for emotion recognition using EEG, GSR, and PPG in response to TEM.

Our proposed methodology to classify human emotions using EEG, GSR, and PPG in response to TEM is shown in Figure 1. There are five phases including content generation, data acquisition, pre-processing, feature extraction and modality level fusion, and classification. Each of these phases is discussed in detail in the following subsections.

### 3.1. TEM Content Generation

TEM was generated for simultaneously engaging three (vision, tactile, and auditory) human senses. Four different video clips were selected, which were then synchronized with a heater and an electric fan. The first clip was selected from the movie *‘Tangled’*, where a character faces opposing effect of air while running on snow. For TEM clip 1 generation, the timestamp (to start the airflow) was identified and synchronized with an electric fan. The second clip was selected from an online source (youtube.com), where a character is seated behind an airplane with the opposing effect of air. The timestamp was identified and synchronized with a fan to generate TEM clip 2. The third clip was selected from the movie ‘*The Lord of the Rings*’, where a character faces the effect of heat generated by a volcano. The timestamp was identified on the basis of unfurling of hair, and synchronization was performed with an electric heater to generate TEM clip 3. The fourth clip was selected from an online source, where a character ignites fire in a cold environment surrounded by snow. The timestamp for this event was identified and synchronized with an electric heater to generate TEM clip 4. Since tactile sensation, in the selected videos, were felt on the face and hands of the character, therefore fan and heater were placed on the right and left side of the viewer respectively. For cold air effect, a DC fan of 8 inches wing size operated at 10 V in full swing was used. For hot air effect, electric fan heater was operated at 240 V and 1000 W.

**Figure 2:**
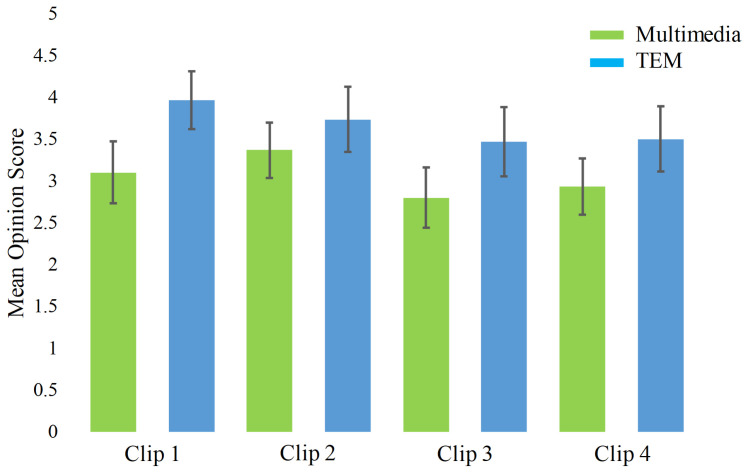
Confidence interval plot of MOS with 95% confidence in response to traditional multimedia and TEM clips.

Five users initially evaluated the synchronization of each clip with fan and heater using the user feedback on a five-point Likert scale. The best synchronization point was identified according to the ratings from these users. The timestamp details of each TEM clip and the total duration of the hot air or cold air effect are shown in Table 2. The audio-visual content was synchronized with electrical fan and heater using timestamp information associated with the help of our own designed TEM player. Moreover, the user experience was evaluated while watching traditional multimedia and TEM. For this purpose, 30 users watched all four multimedia clips and their TEM versions in a random order. The question asked was: *‘Does the tactile effect enhance the sensation of reality while watching the clip?’*. Users rated the experience feedback on a five-point Likert scale from “*Strongly Agree*” at one end to “*Strongly Disagree*” at the other end. Mean opinion scores (MOS) with 95% confidence interval of four clips and their tactile enhanced version is shown in Figure 2. A MOS of 3.1, 3.3, 2.9, and 3.0 against traditional multimedia clip 1, 2, 3, and 4 and 4.0, 3.8, 3.5, and 3.6 against TEM clips 1, 2, 3, and 4 was observed. This suggests that users had a better experience with TEM when compared with the traditional multimedia content. A t-test was also applied to the experience scores of traditional multimedia clips and its TEM versions to identify the significant difference between the two groups. A *p*-value of 0.0001, 0.0453, 0.0063, and 0.0009 was obtained for clip 1, 2, 3, and 4 respectively, which shows a significant difference in the perceived experience.

### 3.2. Data Acquisition

#### 3.2.1. Participants

In this study, a total of 21 participants (10 females and 11 males, average age = 21.1 years) participated voluntarily. It is to be noted here that these participants were different from those who recorded the scaling scores for synchronization and MOS for experiencing the TEM clips. There was no reported history of disability, mental, or physical illness for any of the participants involved. The experimental study was designed according to the Helsinki declaration and approved by the Board of Advanced Studies Research and Technological Development, University of Engineering and Technology, Taxila, Pakistan.

#### 3.2.2. Apparatus

**Figure 3:**
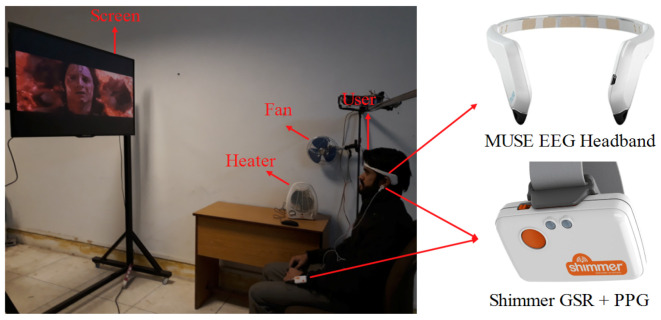
Experimental setup and apparatus used for data recording while watching TEM clips.

The data acquisition setup for experiencing TEM content along with the apparatus is shown in Figure 3. A commercially available EEG headband (Muse) was used for recording EEG signals, whereas a Shimmer GSR and PPG module was used for recording GSR and PPG signals. The Muse headband has two temporal (TP9 and TP10) and two frontal (AF7 and AF8) electrodes, which are designed to be placed according to the international 10–20 electrode positioning system. The GSR electrodes were placed on the hand finger, and PPG electrode was placed on the ear lobe. EEG, GSR, and PPG data were acquired at a sampling rate of 256 Hz.

#### 3.2.3. Experimental Procedure

**Figure 4:**
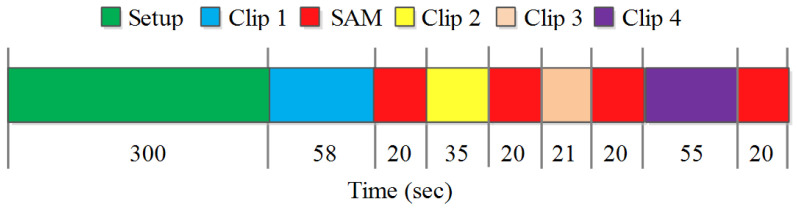
Experimental procedure followed for physiological data acquisition in response to TEM clips.

Each participant was initially briefed about the scope of the experiment. This was followed by signing of a written consent form and demographic details were recorded. The procedural diagram for data acquisition is shown in Figure 4. At the start of the experiment, wearable sensors were set up on an individual’s forehead, fingers, and ear lobe for EEG, GSR, and PPG data recording, respectively. Each TEM clip was then displayed to the participant on a 55 inch LED display. Each participant was provided with a comfortable chair to experience TEM clips in normal room temperature and lighting conditions. The viewer had to rate the clip on a 9-point SAM scale. SAM is a graphical (non-verbal) tool to measure the user’s affective reaction in response to a variety of stimuli in terms of valence, arousal, and dominance [93]. The valence dimension is represented graphically from smiling (happy) figure to frowning (unhappy) figure. Similarly, the arousal dimension is represented graphically from excited (wide-eyed) figure to relaxed (sleepy) figure. In this study, valence and arousal scores were recorded on a paper at the end of each clip (represented as red block in the sequence diagram). The valence value shows the pleasant-unpleasant state, whereas arousal represents the calm-excited state of an individual. In this study, Russell’s Circumplex model [7] was used to label emotions into four groups based on their valence arousal scores. Happy, angry, sad, and relaxed emotions were categorized based on high-valence high-arousal, low-valence high-arousal, low-valence low-arousal, and high-valence low-arousal groups, respectively.

### 3.3. Pre-Processing

The recorded data were pre-processed to remove noise generated by muscular movements or external interference. The time-series signals (including EEG, GSR, and PPG) were filtered using the Savitzky-Golay (SG) filter [94], which was used for smoothing the data without distorting the signal tendency. Moreover, an on-board driven right leg (DRL) feedback circuit on Muse EEG headband canceled the noise present in the EEG signal. The DRL circuits are often used in physiological signal amplifiers to minimize common-mode interference. An on-board signal processing module characterized the data in different frequency (alpha, beta, delta, theta, and gamma) bands. Muscular and eye movement artifacts were also minimized by asking the participants to avoid unnecessary movements during data recording.

### 3.4. Feature Extraction and Modality Level Fusion

After pre-processing, features were extracted from the recorded physiological sensors data to recognize human emotions. Frequency-domain features including rational asymmetry ($RASM_{b}$), differential asymmetry ($DASM_{b}$), and correlation ($C_{b}$) were extracted from each band of the EEG signal. Asymmetry features and correlation were calculated from symmetric electrode pairs from right and left hemispheres (i.e., (AF7,AF8) and (TP9,TP10)) of the brain. These features are related to the valence of an emotional stimulus [95,96]. Four time-domain features were extracted from the recorded GSR data including entropy (*E*), variance (*V*), kurtosis (*K*), and skewness (*S*). Heart rate (HR) and heart rate variability (HRV) were calculated as features from the recorded PPG data. HR was calculated using the number of R-peaks within the signal whereas, HRV was calculated as the average time interval between successive R-peaks.

The extracted EEG, GSR, and PPG features are summarized in Table 3. In total 30, 4, and 2 features were extracted from EEG, GSR, and PPG signals respectively. Hence a total of 36 values were obtained resulting in a feature matrix of size 84×36, where 84 accounts for each of the 21 participants viewing 4 TEM clips. In multimodal emotion recognition frameworks, information from multiple modalities can be fused either using modality level fusion (MLF) or decision level fusion (DLF) [43]. In DLF, multiple classifiers are used on each modality features and their decisions are fused. In MLF, features from different modalities are fused prior to classification. In this study, MLF is selected as fusion technique because single classifier is used as compared to DLF that utilizes multiple classifiers. As in MLF, classification was performed after fusing features from all modalities. Therefore, all possible combinations of features were tested from different modalities (including EEG, GSR, and PPG).

### 3.5. Classification

The classification of four emotions in response to TEM content was performed using the k-nearest neighbor (KNN) algorithm. It has been widely used for emotion recognition in response to different types of stimuli using physiological signals [97]. KNN was chosen because of its inherent simplicity in implementation and robustness to noisy data. Since we are dealing with physiological signals acquired using commercial grade devices, the level of noise could be high, and a robust classifier benefits the classification task. In KNN, the input data are classified on the basis of votes of its neighbors. The training phase in KNN involves a vector in a multidimensional space along with class labels. In the classification phase, a label is assigned to an unlabeled vector and classified on the basis of nearest sample from the training data. We used cross-validation and split the data into test and train samples such that there was no overlap among these sample points.

## 4. Experimental Results and Discussion

### 4.1. Data Labeling

**Figure 5:**
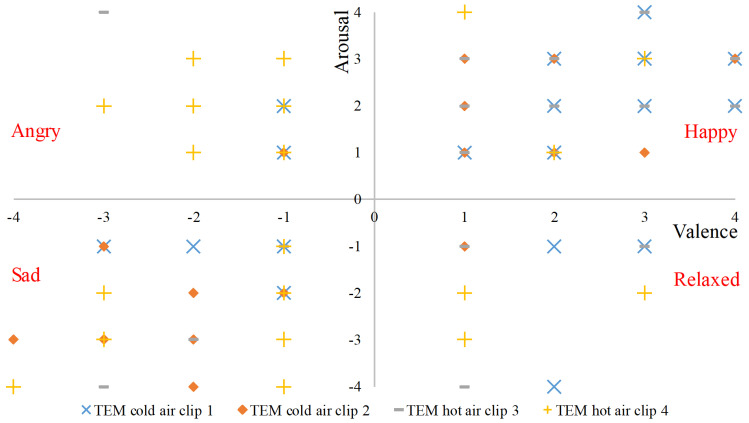
Recorded SAM scores in response to four TEM clips.

Emotions were represented in a 2-dimensional space using valence and arousal scores. The emotion from each quadrant was labeled based on the arousal and valence score recorded in response to each clip. SAM scores for all participants against each TEM clip are shown in Figure 5. Positive arousal and valence scores were labeled as a happy state, whereas negative arousal and valence were tagged as a relaxed state. Similarly, positive valence but negative arousal was labeled as angry emotion whereas, negative valence but positive arousal was labeled as a sad state. The total number of instances labeled as happy, angry, sad, and relaxed emotions were 40, 13, 22, and 9 respectively.

### 4.2. Performance Evaluation

Herein, we present the classification performance of our proposed scheme to classify four emotions (happy, relaxed, angry, and sad) in response to TEM content. A 10-fold cross-validation scheme was applied to train the classifier. Classifier’s performance against each modality was compared in terms of accuracy, squared error rates (root mean squared error (RMSE), root relative squared error (RRSE)), absolute error rates (mean absolute error (MAE), relative absolute error (RAE)), and kappa statistics. The kappa parameter (range: −1<k<1) indicates the agreement of testing data with the training data.

Various parameters for evaluation of emotion classification performance in response to TEM (for EEG, GSR, and PPG) are presented in Table 4. For MLF, various combinations of features from different modalities were used. For individual physiological sensors, we observe that PPG achieved the highest accuracy of 78.57% with a lower absolute and squared error rates as compared to EEG (75.00%) and GSR (72.61%). A high value of kappa (k=0.678) using PPG features also suggests a high inter-rater agreement of testing and training data as compared to EEG and GSR based features. GSR features have the lowest accuracy and kappa value, and higher error rates for emotion recognition. We observe no significant improvement in performance for combinations where features from two modalities were fused. Whereas, fusing features from all three modalities improved the emotion recognition accuracy up to 79.76%. In addition, lower value of absolute and squared error rates and a higher value of kappa (k=0.690) shows better performance of the emotion recognition system using MLF (combining features for EEG, GSR and PPG).

**Figure 6:**
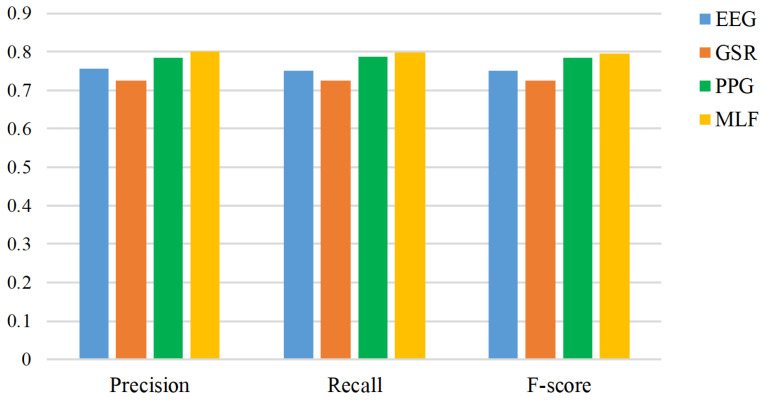
Precision, recall, and F-score in response to TEM using EEG, GSR, PPG, and MLF.

The number of correctly classified and misclassified instances is represented by the confusion matrix (Table 5). Each class was evaluated in terms of sensitivity and specificity. Sensitivity is also known as the true positive rate and measures the proportion of correctly classified instances. Specificity measures the proportion of actual negatives. We observe that the happy emotion has the highest sensitivity as compared to sad, angry, and relaxed emotions. Whereas, angry emotion has the highest specificity as compared to other emotions using EEG. It was also observed that the happy state has the highest sensitivity using PPG as compared to GSR and EEG based features. The happy and angry emotions were correctly classified with the highest sensitivity using fusion as compared to individual modalities. This resulted in the highest accuracy when the proposed emotion recognition system (utilizing modality level fusion of EEG, GSR, and PPG signals) was used with TEM content. The classifier performance was also evaluated in terms of precision, recall, and F-score, which are shown in Figure 6. Here we can observe that the precision, recall, and F-score are higher using MLF (EEG+GSR+PPG) as compared to EEG, GSR, and PPG based features. This is an indicator of better performance using our proposed emotion recognition system with modality level fusion of features.

### 4.3. Discussion

We presented an experimental study to recognize human emotions in response to TEM clips using physiological signals i.e., EEG, GSR, and PPG. From our experimental results, we observed that PPG based features (HR and HRV) could be used to classify human emotions (four) more precisely as compared to EEG and GSR based features. Moreover, it is also observed that the accuracy of emotion recognition in response to TEM clips increases by fusing the features from each of these modalities. Our proposed emotion recognition scheme is compared with state-of-the-art techniques in terms of modality used, the number of emotions, the number of videos, the number of users, and the accuracy of the system as shown in Table 6.

The vibro-tactile content was generated by synchronizing 6 video clips with a haptic vest [91]. This content was intrusive in nature, since physical contact with the human body was required. A haptic vest was used to generate a vibration (synchronized to the scene) producing vibro-tactile effect. The perception and enjoyment of users in response to different vibration settings were statistically analyzed by using a wrist worn heart rate sensor and eye gaze data. These sensors were utilized to explore the cross-modal correspondences and quality of experience of users using different vibration settings. The results were analyzed using subjective questionnaires, however the impact of such enhanced content on human brain activity and emotions was not evaluated. Two clips were synchronized (for cold and hot air effect) with a fan and a heater to generate TEM content [35]. This enhanced content was non-intrusive, since none of the components made any physical contact with the human body. Air was used as a medium to carry the hot and cold air effects to the user. EEG signals were recorded and time-domain features were extracted using Muse EEG headband. Four emotions were classified with an accuracy of 63.41%. Furthermore, frequency-domain features were used to improve the emotion recognition accuracy for the same TEM clips [36]. The importance of different frequency-domain features was highlighted, since an emotion recognition accuracy of 76.19% was achieved. Although an average accuracy of 94.02% was achieved for classifying six basic emotions using video as stimuli [85]. It was identified that this performance was possible using a certain variation of the CNN model, and training such parameter intensive models was a challenge. Similarly, five emotional states were recognized using a fusion of PPG, EMG, and GSR signals using video as stimulus [98]. A maximum accuracy of 89.53% was achieved using deep belief network architecture, although it was time consuming to train the model. Both the studies that show higher accuracy have used videos as stimuli, while we used tactile enhanced multimedia as stimuli that engage three human emotions.

Herein, the TEM content was extended by synchronizing four clips with a fan and a heater. Furthermore, we used signals from three modalities for emotion recognition. This stands out from other state-of-the-art studies where, to the best of our knowledge, only EEG signals have been used to account for physiological responses to TEM content. An accuracy of 75.00% is achieved when only EEG based features were used which is higher than [35] and lower (by 1.19%) than [36]. We argue that this slight decrease in the accuracy is due to an increase in the number of clips. Our proposed emotion recognition system employed a multimodal fusion strategy and achieved a higher accuracy (79.76%) when compared with recently reported methods. Further, the benefit of using TEM content was evident when the classification accuracy for same emotions was evaluated while using videos (without tactile effect) as stimuli. It was observed that the accuracy improved by ≈10% when TEM was used for similar video clips. Although improvement in emotion recognition accuracy is achieved but there are certain aspects that need to be further explored in the future. In this study, electric fan and heater were used to generate cold and hot air effects at fixed intensities. The impact of hot and cold air on human response can be different if the intensities are changed. The aim is to generate a sensation, where a user instantly feels a change in environment (temperature in our experiment), which is synchronized to the video content. While our study shows that with TEM, the emotion recognition accuracy increases, which could mean that the users were able to better feel the emotions as the video content intended to deliver. The use of physiological sensors also ensures that the true sensation of emotion is detected which is subjectively independent of users. Although, the number of subjects involved needs to be increased in future to further strengthen the findings of this study. Moreover, the number of clips should be increased including haptic effects to validate the performance of the proposed emotion recognition technique. In general, we can conclude, that tactile enhanced multimedia content can better invoke emotions in users and for affective computing the emotion detection accuracy can be improved when users are presented with such content.

## 5. Conclusions

In this study, TEM clips were created which would simultaneously entice three human senses including tactile, auditory, and vision. This could improve the emotional experience of a user, which herein was demonstrated by recognizing emotions using physiological signals. Four traditional video clips were selected and synchronized with a heater and an electric fan to engage the tactile sense of the observer in addition to vision and auditory senses. EEG, GSR, and PPG data were acquired in response to these TEM clips. Frequency-domain features from EEG, time-domain features from GSR, and heart rate and heart rate variability from PPG data were extracted. Emotion recognition performance was evaluated to recognize four emotions using the KNN classifier based on features from each modality and their fusion. Our experimental results show that the fusion strategy achieves the highest accuracy of 79.76% with high sensitivity and specificity as compared to individual modalities. It should be noted that there are some limitations presented in the discussion, which should be considered. In particular, the number of participants will be increased in future to further validate the results. We also intend to engage more emotions simultaneously and hence develop systems providing an improved emotional experience when experiencing mulsemedia content.

## Figures and Tables

**Table 1:** A summary of the recent literature on emotion recognition using various stimuli and physiological sensors.

Reference	Stimuli	Senses Engaged	Sensors Used	Emotions Classified	Accuracy
[37]	Music	Auditory	EEG	Happy, sad, love, anger	78.11%
[58]	Music	Auditory	EMG, ECG, GSR, respiration	Low/High valence-arousal	70%
[60]	Images	Vision	GSR, ECG, temperature	Love, joy, surprise, fear	88.33%
[38]	Images	Vision	EEG	Fear	–
[61]	Images	Vision	EEG, peripheral signals	Positively excited, negatively	67%
				excited, calm	
[41]	Images	Vision	GSR, PPG	Low/High valence-arousal	86.7%
[51]	Images	Vision	EEG	Low/ High valence-arousal	62.58%
[71]	Images	Vision	EEG	Low/High valence-arousal	53.72%
[39]	Odors	Olfaction	EEG	Pleasant, unpleasant	99.99%
[40]	Textile Fabrics	Tactile	EEG	Pleasant, unpleasant	70.6%
[42]	Videos	Vision, Auditory	EEG, GSR, EMG, EoG, BVP	Low/High valence-arousal	65%
[63]	Videos	Vision, Auditory	EEG, ECG, EoG, MEG	Low/High valence-arousal	85%
[64]	Videos	Vision, Auditory	EEG, ECG, GSR	Low/High valence-arousal	68%
[66]	Videos	Vision, Auditory	EEG, ECG, GSR, respiration	Joy, funny, anger, fear,	83%
				disgust, neutrality	
[43]	Videos	Vision, Auditory	EEG, Eye	Pleasant, unpleasant, neutral,	76.4%
			Tracking	calm, medium, activated	
[85]	Videos	Vision, Auditory	HRV, respiration	Happiness, fear, surprise,	94%
				anger, sadness, disgust	
[77]	Videos	Vision, Auditory	EEG, GSR	Boredom	79.98%
[78]	Videos	Vision, Auditory	ECG, skin temperature, EDA	Negative emotion	92.5%
[53]	Videos	Vision, Auditory	ECG, EMG, GSR, PPG	Pleasure, fear, sadness, anger	93.42%
[86]	Videos	Vision, Auditory	ECG, skin temperature, EDA	Happiness, surprise, anger,	89%
				disgust, sadness, fear	
[70]	Videos	Vision, Auditory	ECG	Joy, sadness, pleasure, anger,	80%
				fear, neutral	
[72]	Videos	Vision, Auditory	EEG	Amusement, sadness, anger,	60%
				fear, surprise, disgust	
[92]	Videos	Vision, Auditory	EEG	Low, medium, high fear	89.96%
[73]	Videos	Vision, Auditory	EEG	Positive, neutral, negative	68%
[74]	Videos	Vision, Auditory	EEG	Low/High valence-arousal	86.2%
[81]	Videos	Vision, Auditory	EEG, GSR	Low/High valence-arousal	73.4%
[69]	Videos	Vision, Auditory	EEG	Joy, neutrality, sadness,	54.52%
				disgust, anger, fear	
[35]	Tactile enhanced	Vision, Auditory,	EEG	Happy, angry, sad, relaxed	63.41%
	multimedia	Tactile			

**Table 2:** Synchronization timestamp and tactile effect duration of TEM clips used in this study.

TEM	Sensorial	Clip	Synchronization	Duration of
Clip	Effect	Duration	Timestamp	Sensorial Effect
Clip 1	Cold air	58 s	00:19–00:59	40 s
Clip 2	Cold air	35 s	00:03–00:30	27 s
Clip 3	Hot air	21 s	00:12–00:28	16 s
Clip 4	Hot air	55 s	00:30–00:55	25 s

**Table 3:** Description of extracted features in this study for emotion recognition.

Sensor	Feature Description
	$RASM_{b}=\frac{P_{R_{b}}}{P_{L_{b}}}$, where $P_{R_{b}}$ and $P_{L_{b}}$ represent power on right
	and left hemisphere respectively, and *b*
	represents EEG band.
EEG	$DASM_{b}=P_{R_{b}}-P_{L_{b}}.$
	$C_{b}=\frac{\Sigma \left(P_{R_{b}}-\bar{P_{R_{b}}}\right)\left(P_{L_{b}}-\bar{P_{L_{b}}}\right)}{\sqrt{\Sigma {\left(P_{R_{b}}-\bar{P_{R_{b}}}\right)}^{2}\Sigma {\left(P_{L_{b}}-\bar{P_{L_{b}}}\right)}^{2}}}$, where $\bar{P_{R_{b}}}$ and $\bar{P_{L_{b}}}$ are the
	mean of $P_{R_{b}}$ and $P_{L_{b}}$ respectively.
	$V=E[{\left(X-\mu \right)}^{2}]$, where *X* is the row vector consisting of
	GSR data and μ is the mean value.
	E=−∑(p(X)logp(X)), where p(X) is the probability.
GSR	$K=\frac{m_{4}}{V}$, where $m_{4}$ is fourth moment of the GSR data.
	$S=\frac{m_{3}}{m_{3}^{\frac{3}{2}}}$, where $m_{3}$ is third moment of the GSR data.
PPG	HR = Number of beats in a minute.
	HRV = Time interval between heart beats.

**Table 4:** Classification performance for emotion recognition using EEG, GSR, PPG, and modality level fusion in response to TEM content.

Modality	Accuracy	MAE	RMSE	RAE	RRSE	Kappa
EEG	75.00%	0.13	0.35	40.13	84.73	0.62
GSR	72.61%	0.14	0.35	42.68	86.15	0.59
PPG	78.57%	0.11	0.31	33.13	75.18	0.68
EEG+GSR	69.04%	0.13	0.28	39.30	69.54	0.57
EEG+PPG	72.61%	0.13	0.32	37.97	79.25	0.60
GSR+PPG	75.00%	0.12	0.30	35.83	72.93	0.63
EEG+GSR+PPG	79.76%	0.11	0.31	33.23	76.06	0.69

**Table 5:** Confusion matrices for emotion recognition using (a) EEG, (b) GSR, (c) PPG, and (d) MLF (EEG+GSR+PPG) in response to TEM content.

a	b	c	d	Classified as	Sensitivity	Specificity
6	1	2	0	a = Relaxed	66.7%	94.0%
0	15	6	1	b = Sad	68.2%	91.9%
4	2	33	1	c = Happy	82.5%	80.0%
1	2	1	9	d = Angry	69.2%	97.2%
(a) EEG						
7	1	2	1	a = Relaxed	77.8%	94.7%
0	18	5	0	b = Sad	81.8%	91.9%
2	3	29	5	c = Happy	72.5%	77.3%
0	0	4	7	d = Angry	53.8%	94.4%
(b) GSR						
7	0	4	0	a = Relaxed	77.8%	94.7%
0	17	1	4	b = Sad	77.3%	91.9%
2	2	35	2	c = Happy	87.5%	86.4%
0	3	0	7	d = Angry	53.8%	95.8%
(c) PPG						
7	0	1	0	a = Relaxed	77.8%	98.7%
1	16	3	2	b = Sad	72.7%	90.3%
1	6	35	2	c = Happy	87.5%	79.5%
0	0	1	9	d = Angry	69.2%	98.6%
(d) EEG+GSR+PPG						

**Table 6:** Performance comparison of the proposed emotion recognition system in response to TEM with state-of-the-art methods.

Method	Modality	Emotions	No. of	No. of	Accuracy
			TEM/Video Clips	Users (F/M)	
[91]	Eye gaze, Heart	Enjoyment, Perception	6 (TEM)	24 (9/15)	-
	rate wrist band				
[35]	EEG	Happy, Angry, Sad, Relaxed	2 (TEM)	21 (10/11)	63.41%
[36]	EEG	Happy, Angry, Sad, Relaxed	2 (TEM)	21 (10/11)	76.19%
[85]	RSP and HRV	Happy, Angry, Sad, Fear, Surprise, Disgust	6 (video)	49 (19/30)	94.02%
[98]	PPG, EMG, EDA	Happy, Sad, Disgust, Relaxed, Neutral	40 (video)	32 (16/16)	89.53%
Proposed	EEG, GSR, PPG	Happy, Angry, Sad, Relaxed	4 (Video)	21 (10/11)	70.01%
Proposed	EEG	Happy, Angry, Sad, Relaxed	4 (TEM)	21 (10/11)	75.00%
Proposed	EEG, GSR, PPG	Happy, Angry, Sad, Relaxed	4 (TEM)	21 (10/11)	79.76%
